# Data for spatial characterization of AC signal propagation over primary neuron dendrites

**DOI:** 10.1016/j.dib.2015.12.018

**Published:** 2015-12-17

**Authors:** Hojeong Kim, C.J. Heckman

**Affiliations:** a Division of IoT·Robotics Convergence Research, DGIST, Daegu, Republic of Korea; b Department of Physiology, Northwestern University, Chicago, USA; c Department of Physical Medicine and Rehabilitation, and Physical Therapy and Human Movement Science, Northwestern University Feinberg School of Medicine, Chicago, USA

**Keywords:** Primary neurons, Dendritic signal processing, AC signal propagation, Voltage attenuation analysis

## Abstract

Action potentials generated near the soma propagate not only into the axonal nerve connecting to the adjacent neurons but also into the dendrites interacting with a diversity of synaptic inputs as well as voltage gated ion channels. Measuring voltage attenuation factors between the soma and all single points of the dendrites in the anatomically reconstructed primary neurons with the same cable properties, we report the signal propagation data showing how the alternating current (AC) signal such as action potentials back-propagates over the dendrites among different types of primary neurons. Fitting equations and their parameter values for the data are also presented to quantitatively capture the spatial profile of AC signal propagation from the soma to the dendrites in primary neurons. Our data is supplemental to our original study for the dependency of dendritic signal propagation and excitability, and their relationship on the cell type-specific structure in primary neurons (DOI: 10.1016/j.neulet.2015.10.017 [1]).

## Specifications Table

**Table:** 

Subject area	Biology
More specific subject area	Computational neurophysiology/Dendritic signal processing/Dendritic computations
Type of data	Figure
How data was acquired	Anatomical reconstruction of primary neurons in the central nervous system, voltage attenuation analysis along individual paths of the dendrites from the soma, least-squares fitting method
Data format	Raw and processed
Experimental factors	Primary neurons with representative morphologies, alternating current (AC) input with the frequency of 250 Hz, voltage attenuation factors between the soma and all single points of the dendrites for the AC input to the soma
Experimental features	Primary neurons were anatomically reconstructed and analyzed in the passive mode under the NEURON software environment.
Data source location	DGIST, Daegu, Korea
Data accessibility	Data is with this article

## Value of the data

• We have spatially characterized the dendritic signal propagation that is a fundamental property underlying dendritic computations and neuronal dynamics.
• The data presented here provide an important information on the relationship between dendritic structure and signal propagation in primary neurons.
• The data measured in the passive dendrites may serve as a basis for interpreting the active back-propagation of action potentials in the dendrites.
• Fitting equations for the data can be used for realistically building the reduced models as a function of the path length from the soma.

## 1 Data

**Fig. 1:**
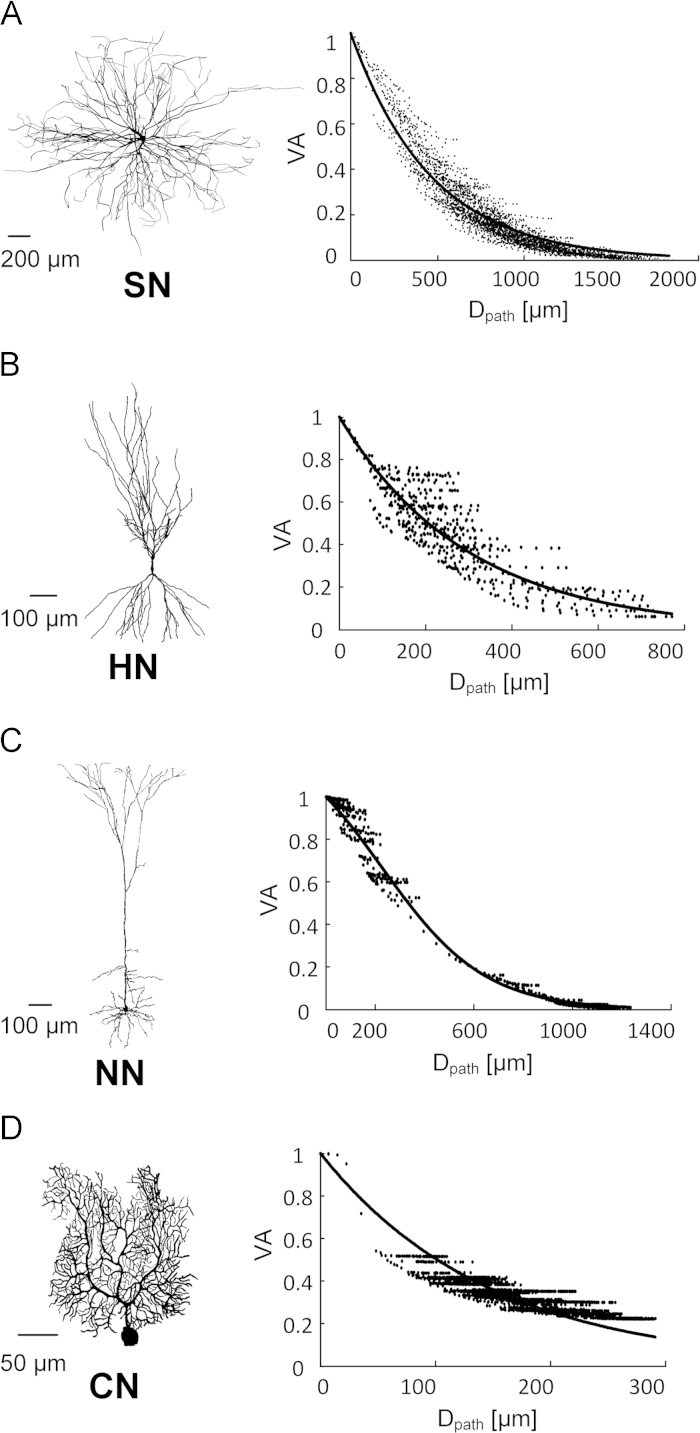
Voltage attenuation factors from the soma to the dendrites with AC input to the soma (${\mathrm{VA}}_{\mathrm{SD}}^{\mathrm{AC}}$)**.** (A)–(D): Morphologies of primary neurons (Spinal motor neuron (SN), hippocampal pyramidal neuron (HN), neocortical pyramidal neuron (NN), and cerebellar Purkinje neuron (CN) from top to bottom) in the left column and the ${\mathrm{VA}}_{\mathrm{SD}}^{\mathrm{AC}}$ data (black dots) at all distances (*D*_path_) from the soma and fitting curves (solid black lines) in the right column.

The spatial profile of voltage attenuation factors (${\mathrm{VA}}_{\mathrm{SD}}^{\mathrm{AC}}$) measured between the soma and all individual points of the dendrites while applying a 250 Hz AC input to the soma for each type of anatomically reconstructed primary neurons with representative morphologies (spinal motor neuron, hippocampal pyramidal neuron, neocortical pyramidal neuron and cerebellar Purkinje neuron). A single exponential function (exp(−*D*_path_/*λ*)) was used to fit the ${\mathrm{VA}}_{\mathrm{SD}}^{\mathrm{AC}}$ (*λ*=464.7 for the spinal motor neuron, 297.72 for the hippocampal pyramidal neuron and 146.51 for the cerebellar Purkinje neuron). The ${\mathrm{VA}}_{\mathrm{SD}}^{\mathrm{AC}}$ data in the neocortical pyramidal neuron were best fit to the modified Boltzmann equation (1/[1−exp(−*α*_1_/*α*_2_)+exp((*D*_path_−*α*_1_)/*α*_2_)]), where *α*_1_=287.16 and *α*_2_=209.38 Fig. 1.

## 2 Experimental design, materials and methods

To investigate the relationship between the dendritic structure and signal propagation in primary neurons [1], the data for the representative morphology of a rat neocortical L5 pyramidal cell (p21 in Dendritica), a rat hippocampal CA3 pyramidal cell (2189201 in Claiborne׳s lab), a rat cerebellum Purkinje cell (p19 in Dendritica), and a cat spinal α-motoneuron (Vemoto6 in Burke׳s lab) were downloaded directly from http://NeuroMorpho.Org [2] and translated into the NEURON simulation environment v 6.1.1 using the Import3D tool [3]. To isolate the effect of the dendritic structure, the same values of cable parameters that were experimentally identified from the spinal motoneuron were assigned to all anatomically reconstructed primary neurons (*R*_m,soma_=225 Ω cm^2^, *R*_m,dendrite_=1100 Ω cm^2^, *C*_m_=1 μF/cm^2^, and *R*_a_=70 Ω cm, [4]). The selection of cable parameter values from the spinal motoneuron was made such that the models for primary neurons in the brain maintained the lowest input resistance (*R*_N_) at their somata.

For the analysis of dendritic signal propagation in the anatomically reconstructed primary neurons, a voltage attenuation (VA) factor was defined as the ratio of the voltage amplitude at the measurement site to the voltage amplitude at the stimulation site [5] and calculated using the Impedance class tools built in the NEURON software. The ${\mathrm{VA}}_{\mathrm{SD}}^{\mathrm{AC}}$ data from the soma to the dendrites were calculated between the soma and all individual points in the dendrites in response to a 250 Hz alternating current (AC) input to the soma. The frequency of AC input was calculated from the period of 4 ms assuming the width of an action potential to be 2 ms. The fitting equation and parameter values for the ${\mathrm{VA}}_{\mathrm{SD}}^{\mathrm{AC}}$ data were identified using the method of least squares that was built in the MATLAB software.
